# Modeling of anisotropic properties of double quantum rings by the terahertz laser field

**DOI:** 10.1038/s41598-018-24494-w

**Published:** 2018-04-18

**Authors:** Henrikh M. Baghramyan, Manuk G. Barseghyan, Albert A. Kirakosyan, Judith H. Ojeda, Jean Bragard, David Laroze

**Affiliations:** 1 Instituto de Alta Investigación, CEDENNA, Universidad de Tarapacá, Casilla 7D, Arica, Chile; 2 Armenian State Pedagogical University after Khachatur Abovyan, Tigran Mets ave. 17, Yerevan, 0010 Armenia; 3 Department of Solid State Physics, Yerevan State University, Alex Manoogian 1, 0025 Yerevan, Armenia; 4 National University of Architecture and Construction of Armenia, Teryan 105, 0009 Yerevan, Armenia; 5 Grupo de Física de Materiales, Escuela de Física, Universidad Pedagógica y Tecnológica de Colombia, Tunja, Colombia; 6 Departamento de Física y Matemática Aplicada - Universidad de Navarra -, 31080 Pamplona, Spain; 7 School of Physical Sciences and Nanotechnology, Yachay Tech University, 00119 Urcuquí, Ecuador

## Abstract

The rendering of different shapes of just a single sample of a concentric double quantum ring is demonstrated realizable with a terahertz laser field, that in turn, allows the manipulation of electronic and optical properties of a sample. It is shown that by changing the intensity or frequency of laser field, one can come to a new set of degenerated levels in double quantum rings and switch the charge distribution between the rings. In addition, depending on the direction of an additional static electric field, the linear and quadratic quantum confined Stark effects are observed. The absorption spectrum shifts and the additive absorption coefficient variations affected by laser and electric fields are discussed. Finally, anisotropic electronic and optical properties of isotropic concentric double quantum rings are modeled with the help of terahertz laser field.

## Introduction

Modern solid-state physics encompasses activities far beyond the subject of conventional bulk semiconductors, involving the design, fabrication, study, and applications of the broad range of nanostructures. Among them, quantum rings (QRs)^1^, occupy an outstanding place, because they are non-simply connected zero-dimensional coherent clusters of atoms or molecules on a surface^2^, which makes them ideal structures for the study of topological quantum mechanical phenomena, like Aharonov-Bohm effect^3,4^. The confinement in these nanostructures is stronger than in quantum dots (QDs) owing to the altered and multiply connected shape, that can result in a single bound state and be suitable for terahertz (THz) intersublevel detectors with a strong response in the 1–3 THz range^5^. The tunneling effect in QRs is responsible for the intermediate-band in the coupled array of QRs^6^. It was used as an additional path for the electron transitions to the continuum to enhance the photocurrents for solar cell applications^7^ and resonant tunneling devices as well^8^. Besides, doubly-connected ring-like geometry is currently used to form materials with unique properties: in quantum dot-ring nanostructures (a QD surrounded by a QR)^9,10^ spin relaxation times, optical absorption and conducting properties are highly tunable by means of the confinement^11,12^; electrochemical performance and structure evolution of core-shell nano-ring *α*-Fe_2_O_3_*@*Carbon anodes for lithium-ion batteries^13^; the electrical properties of a p-type semiconductor can be mimicked by a metamaterial solely made up of an n-type semiconductor ZnO rings^14^; etc.

More importantly, the assembly of concentric double quantum rings (CDQRs)^15^ is especially interesting, in the light of the coupling between the rings. In fact, the electronic transport through the outer ring of a CDQR device showed oscillations with two distinct components with different frequencies, which were caused by the Aharonov-Bohm effect in the outer ring and also attributed to the Coulomb-coupled influence of the inner ring^16^. Moreover, photoluminescence emissions originating from the outer ring and that from the inner ring are observed distinctly^17^. In this geometry, the intensity time-correlation measurements^18^ showed that while the inner ring satisfies the requirement of a quantum emitter of single photons, in the outer ring this requirement is not fulfilled. A recent study by Hofmann *et al*.^19^ experimentally demonstrated the measuring of quantum state degeneracies in bound state energy spectra. Their method is realized using a GaAs/AlGaAs QD allowing for the detection of time-resolved single-electron tunneling with a precision enhanced by feedback control. These experimental results claim the need to investigate how the coupling between the rings in CDQRs can be influenced both externally and internally. Internally, it has been demonstrated to be realizable by varying the inter-ring distance and aluminium concentration during the calculations of few-electron^20^ and impurity-related linear and non-linear optical absorption spectrum^21^ respectively, while externally it can be done by applying magnetic and electric fields^22–27^ and with hydrostatic pressure^28^ as well.

A few works of our group were devoted to the study of intense laser field effects in quantum ring structures^29–33^. The current work aims to demonstrate theoretically that inter-ring coupling in semiconductor concentric double quantum rings can be controlled by intense THz laser field and uniform static electric fields. In particular, it is shown that the laser field can rearrange the energy spectrum by eliminating and afterward creating new pairs of degenerated levels, while the static electric field effect in anisotropic laser-dressed confining potential can create both linear and quadratic Stark effects.

## Problem

The CDQRs consists of GaAs QRs (well material) separated by Ga_0.7_Al_0.3_As (barrier material). In the absence of laser field the confinement of electron in two-dimensional CDQR structure is modeled according to potential:

1 $$V({{\bf{r}}}_{\perp })=\{\begin{array}{l}0,\,{\rm{if}}\,{r}_{\perp }\in [{R}_{1}^{{\rm{in}}},{R}_{2}^{{\rm{in}}}]\cup [{R}_{1}^{{\rm{out}}},{R}_{2}^{{\rm{out}}}]\\ {V}_{0},\,{\rm{elsewhere}},\end{array}$$

where *V*_0_ = 257 meV is the height of the potential attributed to the confinement of electrons^34^, ***r***_⊥_ denotes the electron position in two-dimensional CDQR and ${R}_{1}^{{\rm{in}}},{R}_{2}^{{\rm{in}}},{R}_{1}^{{\rm{out}}}$, and ${R}_{2}^{{\rm{out}}}$ are respectively inner (subscript “1”) and outer (subscript “2”) radii of inner (superscript “in”) and outer (superscript “out”) rings. The rings are considered two-dimensional based on the much stronger quantization in growth direction^17^, and the radii of rings are taken in accordance with the sizes of real CDQR structure^15^. It is also supported by works that used two-dimensional models of confinement to make comparisons with experimental data. In ref.^3^ authors compared the Aharonov-Bohm oscillations in InAs/GaAs QRs using the two-dimensional parabolic confinement. In addition, although the confinement potential in ref.^17^ was defined using the actual shape of CDQRs determined by the atomic force microscopy measurements, mainly the quantized radial motion was considered for the effective mass calculations to compare with photoluminescence data.

In the presence of static electric and THz laser fields the radial and rotational motion of the electron in two-dimensional CDQR can be described by a time-dependent Schrödinger equation:

2 $$[\frac{1}{2m}{({\hat{{\bf{p}}}}_{\perp }-\frac{e}{c}{{\bf{A}}}_{\perp }({\bf{r}},t))}^{2}+V({{\bf{r}}}_{\perp })-e{\bf{F}}\cdot {{\bf{r}}}_{\perp }]\times {\rm{\Phi }}({{\bf{r}}}_{\perp },t)=i\hslash \frac{\partial }{\partial t}{\rm{\Phi }}({{\bf{r}}}_{\perp },t),$$

where *m* = 0.067 *m*_0_ is the effective mass of electron in GaAs^35^, such that *m*_0_ is the rest masses of electron, vector potential **A**_⊥_(**r**, *t*) defines the laser field, *e* denotes the electron charge, *c* is the speed of light, **F** is the electric field strength, and *ħ* is the reduced Planck constant.

The solution of Eq. (2) can be greatly simplified under dipole approximation i.e. when **A**_⊥_(**r**, *t*) ≈ **A**(*t*)^36^. For 10 nm wide (in the radial direction) GaAs QRs, it is fulfilled if the laser field frequency $\nu \ll 1500\,\,{\rm{THz}}$, and in the current work we will deal with such frequencies. With satisfied dipole approximation, vector potential does not vary in space and the phase-factor transformation^37^

3 $${\rm{\Phi }}({{\bf{r}}}_{\perp },t)={\rm{\Psi }}({{\bf{r}}}_{\perp },t)\times \exp [-({\rm{i}}{e}^{2})/(2\hslash m{c}^{2}){\int }^{t}{{\bf{A}}}^{2}(t^{\prime} )dt^{\prime} ]$$

can be applied removing the term with **A**^2^ from Eq. (2):

4 $$[\frac{{\hat{{\bf{p}}}}_{\perp }^{2}}{2m}-\frac{e}{mc}{\bf{A}}\cdot {\hat{{\bf{p}}}}_{\perp }+V({{\bf{r}}}_{\perp })-e{\bf{F}}\cdot {{\bf{r}}}_{\perp }]\times {\rm{\Psi }}({{\bf{r}}}_{\perp },t)={\rm{i}}\hslash \frac{\partial }{\partial t}{\rm{\Psi }}({{\bf{r}}}_{\perp },t\mathrm{).}$$

Moreover, instead of working with Eq. (4) the space-translated version of it can be obtained performing unitary transformation with the translation operator^37^ $\hat{U}=\exp [(i/\hslash ){\boldsymbol{\alpha }}\cdot {\hat{{\bf{p}}}}_{\perp }]$, where ***α***(*t*) = −*e*/(*mc*)${\int }^{t}A(t^{\prime} )dt^{\prime}$ vector is related to the quiver motion of the electron in the laser field. The new wave function $\varphi ({{\bf{r}}}_{\perp },t)=\hat{U}{\rm{\Psi }}({{\bf{r}}}_{\perp },t)$ satisfies the following Schrödinger equation^30,38^:

5 $$[\frac{{\hat{{\bf{p}}}}_{\perp }^{2}}{2m}+V({{\bf{r}}}_{\perp }+{\boldsymbol{\alpha }})-e{\bf{F}}\cdot ({{\bf{r}}}_{\perp }+{\boldsymbol{\alpha }}(t))]\times \varphi ({{\bf{r}}}_{\perp },t)=i\hslash \frac{\partial }{\partial t}\varphi ({{\bf{r}}}_{\perp },t\mathrm{).}$$

In this work, we are interested in the solution of Eq. (5) in the high-frequency limit $\nu \tau \gg 1$, where *τ* is the characteristic transit time of the electron in the structure. It can be obtained by applying the non-perturbative Floquet theory and subsequently keeping only the zero-order terms of Fourier expansions of confinement potential *V*(**r**_⊥_) and wave function *ϕ*(**r**_⊥_, *t*)^39^. These approximations lead to the following time-independent Schrödinger equation^33^:

6 $$[\frac{{\hat{{\bf{p}}}}_{\perp }^{2}}{2m}+{V}_{{\rm{d}}}^{{\rm{F}}}({{\bf{r}}}_{\perp })]{\psi }_{{\rm{d}}}({{\bf{r}}}_{\perp })={E}_{{\rm{d}}}{\psi }_{{\rm{d}}}({{\bf{r}}}_{\perp }\mathrm{).}$$

In Eq. 6 the laser field is considered with fixed linear polarization along the *x*-axis that results in ${V}_{{\rm{d}}}^{{\rm{F}}}({{\bf{r}}}_{\perp })={T}^{-1}\ast {\int }_{0}^{T}V(x+\alpha (t),y)dt-e{\bf{F}}\cdot {{\bf{r}}}_{\perp }$ laser-dressed and electric field influenced effective potential in Eq. (6) and *α*(*t*) = −*α*_0_sin(2*πt*/*T*) is the quiver displacement where *T* is the laser field period. From now on, the peak value ${\alpha }_{0}=-\,(e/m{\varepsilon }_{{\rm{h}}}^{\mathrm{1/4}}{\nu }^{2})\sqrt{I/(2c{\pi }^{3})}$ will be taken to characterise the laser field effect, where *ε*_h_ = 10.9 is the high-frequency dielectric constant in GaAs^35^, and the intensity *I* and *ν* frequency of laser field are in orders of 1 kW/cm^2^ and 1 THz, respectively.

**Figure 1:**
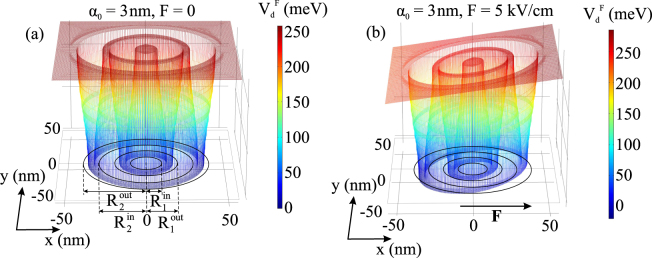
Laser-dressed three-dimensional confining potential for *F* = 0 (**a**) and affected by *F* = 3 kV/cm electric field strength (**b**) The parameter *α*_0_ = 3 nm is fixed and electric field is directed along the *x*-axis.

In Fig. 1 the effective potential ${V}_{{\rm{d}}}^{{\rm{F}}}({{\bf{r}}}_{\perp })$ is presented for the fixed value of *α*_0_ and two different values of electric field strength *F*. While the electric field results in the tilting of the potential, the laser field decreases the width of well regions along the *x*-axis in the lower part of the potential and enlarges them in the upper one. In other words, the laser field creates an anisotropy in the confinement potential, which can be continuously controlled by the THz laser field. It is useful to compare our results with those for elliptic core–multishell quantum wires^40^ and CDQRs^41^. In these works, the anisotropy was induced by the geometry of the structure, that needs to be controlled during the growth process^40^ or by effective mass^41^ manipulations. We theoretically demonstrate (also see Fig. 3 for wave functions) the feasibility of it by THz laser field, that is an external influence. The latter effect allows the investigation of the physical properties of quantum rings of different geometries in a single sample of CDQRs. Thus, our results allow the manipulation of different shapes that is important for modeling of experimental studies of CDQRs that in general are not purely circular^42^.

In addition, we are interested in intraband transitions to estimate the optical response of the laser-dressed system. For that reason, the total absorption coefficient is calculated^43^

7 $$\alpha ({\rm{\Omega }})=A\times \hslash {\rm{\Omega }}\sum _{f}{N}_{{\rm{if}}}{|{M}_{{\rm{if}}}|}^{2}\frac{{\rm{\Gamma }}}{{(\hslash {\rm{\Omega }}-{{\rm{\Delta }}}_{{\rm{fi}}})}^{2}+{{\rm{\Gamma }}}^{2}},$$

where Ω is the incident light angular frequency, ${{\rm{\Delta }}}_{{\rm{fi}}}={E}_{d}^{{\rm{f}}}-{E}_{d}^{{\rm{i}}}$ is the energy difference between the final (*f*) and initial (*i*) states, *M*_if_ defines the dipole matrix element, the Lorentzian parameter is taken equal to Γ = 0.1 meV, and A contains all the other factors^44^. *N*_if_ = *N*_i_ − *N*_f_ is the occupation difference of the ground and final states and is equal to 1, since the final state is vacant and the initial one is the ground state occupied with one electron. Circularly polarized light is considered falling perpendicularly to the plane of the rings.

## Methods

The laser-dressed eigenvalues *E*_d_ and eigenvectors *ψ*_d_(**r**_⊥_) are found numerically in COMSOL Multiphysics software^45^, using the finite element method. Meshing is done with triangular elements, and Lagrangian shape functions are used^46^. A square is taken as a computational domain with side size of $L=2.8{R}_{2}^{{\rm{out}}}$. This value is found sufficient to avoid eigenfunction traces outside of it. In the presence of the fields, the fourth order Lagrangian shape functions are used, and the domain is meshed with *“Extremely fine”* option of *“General physics”* calibration node. In the absence of the fields, third order Lagrangian shape functions and *“Extra fine”* option^45^ is used.

## Results and Discussion

### Degenerated laser-dressed energy spectrum and intraband absorption

**Figure 2:**
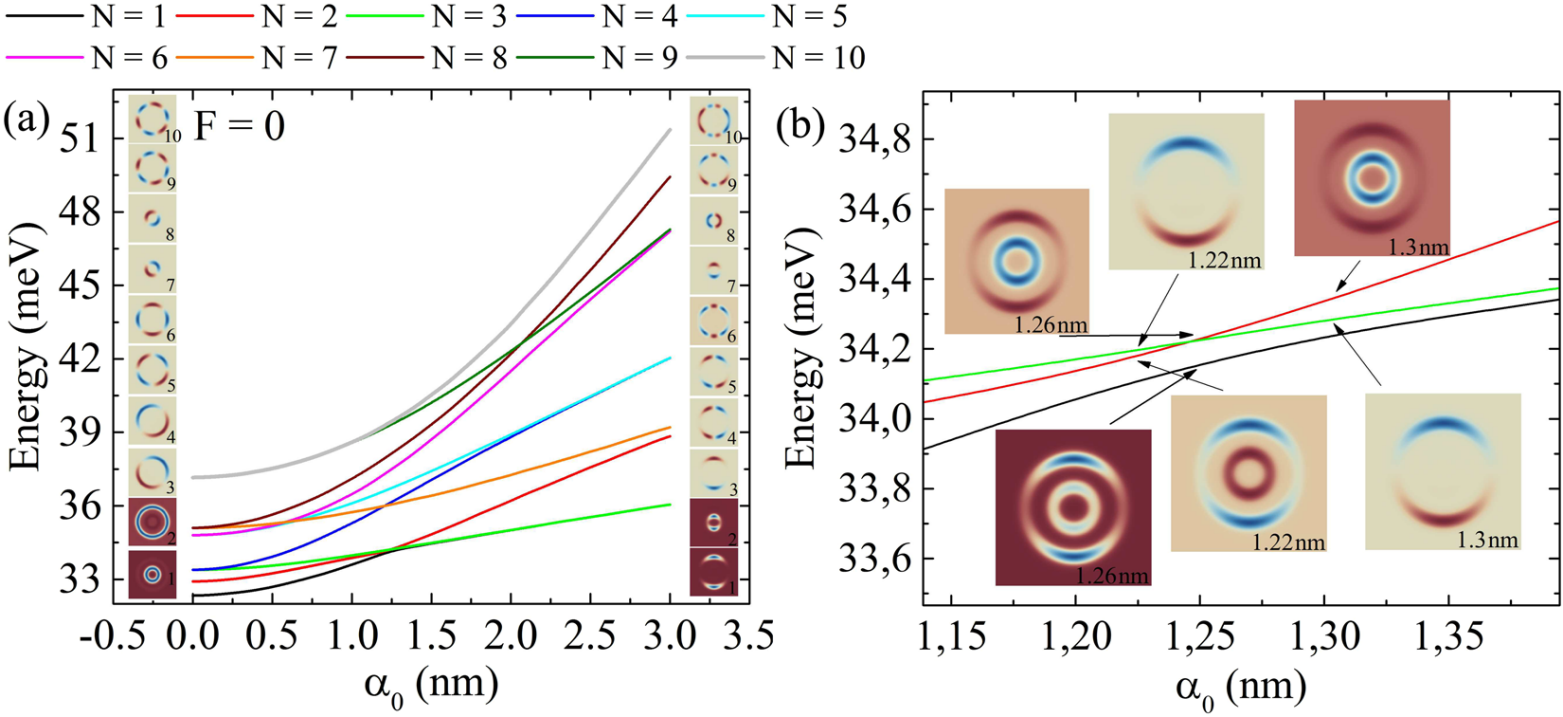
(**a**) laser-dressed energy levels dependence on *α*_0_ in the absence of electric field, where the insets are the wave functions for the considered smallest and biggest values of *α*_0_. (**b**) crossing and anti-crossing points between the first three levels.

Figure 2 illustrates the influence of the laser field on the energy spectrum in the absence of electric field. The inset columns are the wave functions of bound states for the lowest *α*_0_ = 0 and highest *α*_0_ = 3 nm values of laser field parameter. The deformation of the confinement potential ${V}_{{\rm{d}}}^{F}({{\bf{r}}}_{\perp })$ in Fig. 1(a) brings up all the energy levels with the augmentation of *α*_0_, meaning that all the considered ten energy levels are positioned lower enough in the confining potential. Another influence of the laser field is the rearrangement of energy levels: at first, it eliminates the original degeneracy caused by the cylindrical symmetry of confining potential and then it makes new pairs of degenerated levels. In the absence of laser field, the following pairs form degenerated couples: third and fourth, fifth and sixth, seventh and eighth, and ninth and tenth. Viewing the forms of wave functions in Fig. 2(a) at *α*_0_ = 3 nm, one sees that laser field leads to new combinations: first and third, fourth and fifth, and sixth and ninth (second and seventh pair is not that close to like other pairs, but their wave functions have forms similar enough to consider them as ones having a tendency to degenerate afterwards). The reason is the laser field that changes the symmetry axes from the diagonal of the square to the *x* and *y*-axes seen, resulting from the shape modification of confining potential observed in Fig. 1. Also the energy spectrum in Fig. 2(a) is full of crossing points and has only one anti-crossing event, shown in the enlarged graph in Fig. 2(b). For example, the first excited state wave function is symmetric with respect to the *y*-axis, while the third one shows antisymmetry, which means that they can cross, much like the terms of the diatomic molecule^47^. Meanwhile, anti-crossing occurs between the ground and the first excited level, because in the case of crossing the ground state must have zeros, that is not allowed^48^.

**Figure 3:**
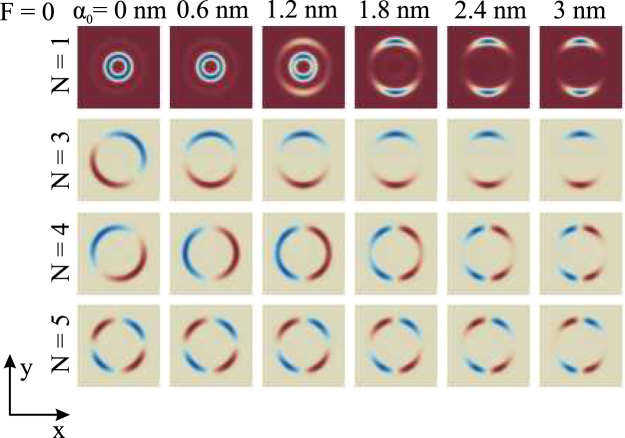
Evolution of the shapes of the first, third, fourth and fifth states with *α*_0_.

The evolution of wave function shapes with the increment of *α*_0_ is depicted in Fig. 3 for the first, third, fourth and fifth states. As expected from the results in Fig. 1, the distribution of wave functions is anisotropic. It localizes along the *y*-axis, as *α*_0_ is increased. Besides, the ground state wave function gradually moves from the inner ring to the outer one and the wave functions show the tendency to accumulate along the *y*-axis. The point is that for low lying states the contraction of the well width is the biggest along the polarization direction of laser field (*x*-axis) and is almost unchanged along the *y*-axis, where the probability to find the electron turns bigger. The obtained modification of electron localization between the rings can be useful for the manipulation transport properties of QR arrays in optoelectronic devices: for example, the proper value of *α*_0_ can shift the electronic cloud to the outer (inner) ring, thus turning on (off) the tunneling between the rings.

**Figure 4:**
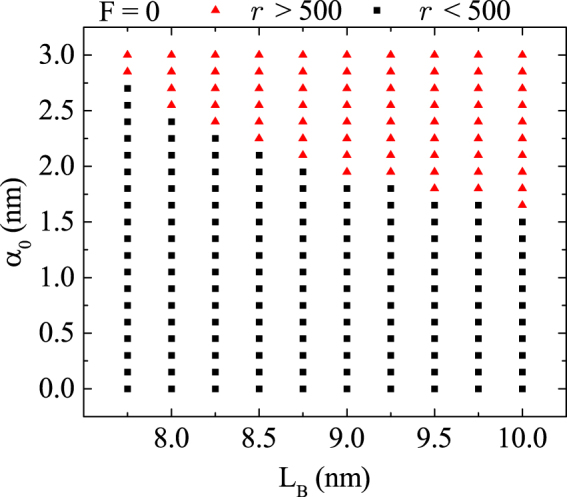
PD delocalization points for different values of *L*_B_ and *α*_0_ when *F* = 0.

In order to study the possibility of the charge delocalization by the THz laser field in CDQR structure, it is interesting to investigate laser field influence on electron probability density (PD) distribution for the ground state by varying the barrier region width *L*_B_ between the inner and outer ring and *α*_0_ at the same time. For that reason, the ratio of probability densities in the outer ring and the inner ring - *r* = (∫_*outer*_|*ψ*_d_|^2^d**r**_⊥_)/(∫_*inner*_|*ψ*_d_|^2^d**r**_⊥_) is calculated. The PD is considered to be fully delocalized to the outer ring, once the *r* > 5 × 10^2^. Under this condition, the map of PD delocalization points is presented in Fig. 4. It can be observed that smaller values of *L*_B_ require bigger ones for *α*_0_ to reach delocalization, and vice versa. The reason for this lays in the coupling of the QRs, which is stronger if the QRs are closer. In addition, the values of *r* at which delocalization occurs do not depend on any fixed ratio *L*_B_/*α*_0_.

**Figure 5:**
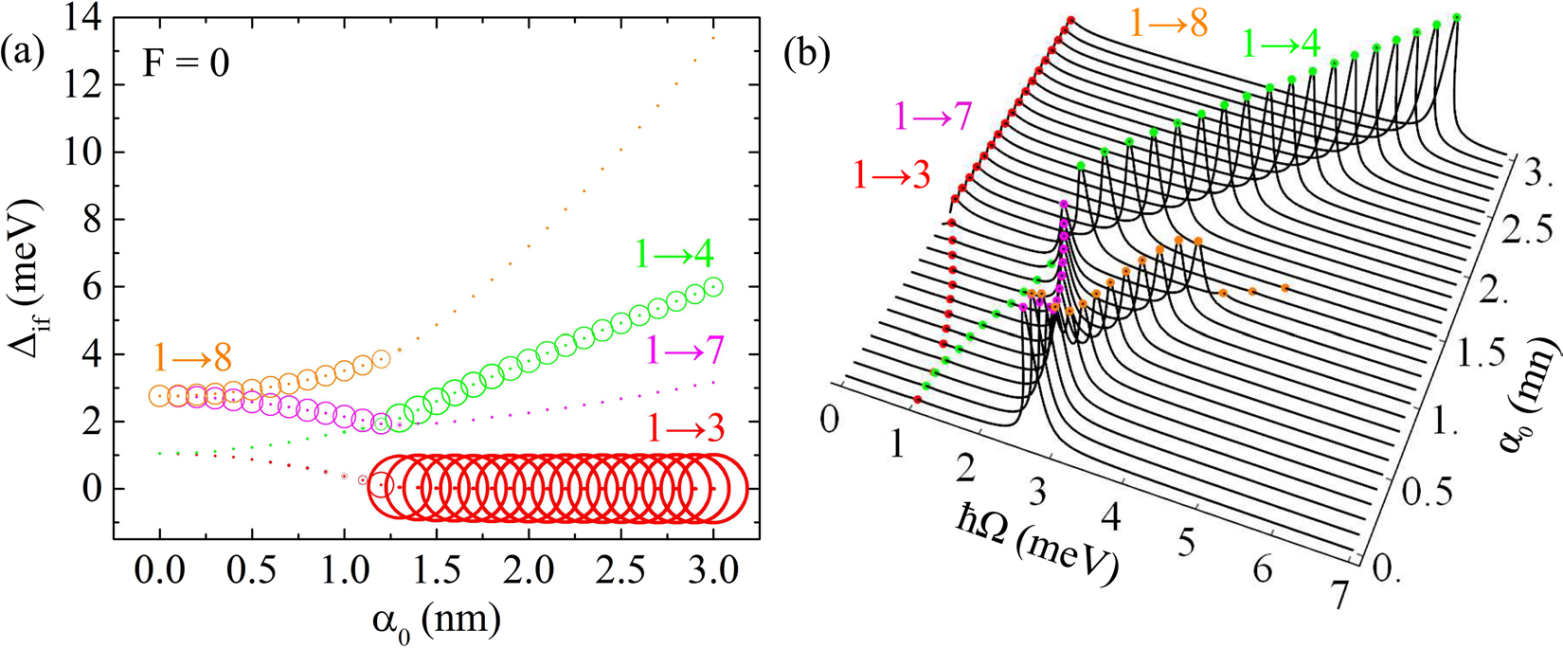
Δ_if_ energy difference dependence on *α*_0_ (**a**) for *F* = 0 and corresponding absorption coefficient (in arbitrary units) dependence on incident photon energy $\hslash {\rm{\Omega }}$ for different values of *α*_0_ (**b**). The area of the circles in Fig. 5(a) is proportional to the respective |*M*_if_|^2^ of the transition.

The Δ_if_ energies dependence on *α*_0_ are shown in Fig. 5(a), where the area of the circles is directly proportional to the dipole matrix element modulus square. The corresponding *α*(Ω) absorption coefficient dependence on incident photon energy $\hslash {\rm{\Omega }}$ by gradually changing values of *α*_0_ is shown in Fig. 5(b) in the absence of electric field *F* = 0. The allowed transitions are 1 → 3, 1 → 4, 1 → 7, 1 → 8, 1 → 9, 1 → 10. The results for 1 → 9 and 1 → 10 are not presented, since their contribution is much weaker compared with other transitions. The selection rule that defines this transitions is based on the symmetry of wave functions of the excited states, that must not have antisymmetry or symmetry with respect to both of the coordinate axes; otherwise the *M*_if_ matrix element is zero. The Δ_if_ curves of 1 → 3 and 1 → 4, 1 → 7 and 1 → 8 pairs start from the same value. It is an expected result, as long as in the absence of laser field the mentioned states are degenerated. Starting from the *α*_0_ = 1.3 nm the 1 → 3 transition has the biggest value of dipole matrix element modulus. Nevertheless, this very issue does not make the maximum of the related absorption coefficient the biggest. Figure 5(b) demonstrates that 1 → 4, 1 → 7 and 1 → 8 transitions have absorption coefficients greater than 1 → 3, although related |*M*_if_|^2^ is much smaller. This is a consequence of $\hslash {\rm{\Omega }}$ factor in Eq.(7). Besides, 1 → 3 shows the redshift, 1 → 4, 1 → 8 transitions undergo a blueshift, and 1 → 7 one in the [0, 1.3 nm] interval demonstrates redshift and subsequently only the blueshift of the absorption spectrum. These spectrum shifts are caused by the results for Δ_if_ as shown in Fig. 5(a).

### Stark effect in laser-dressed states

**Figure 6:**
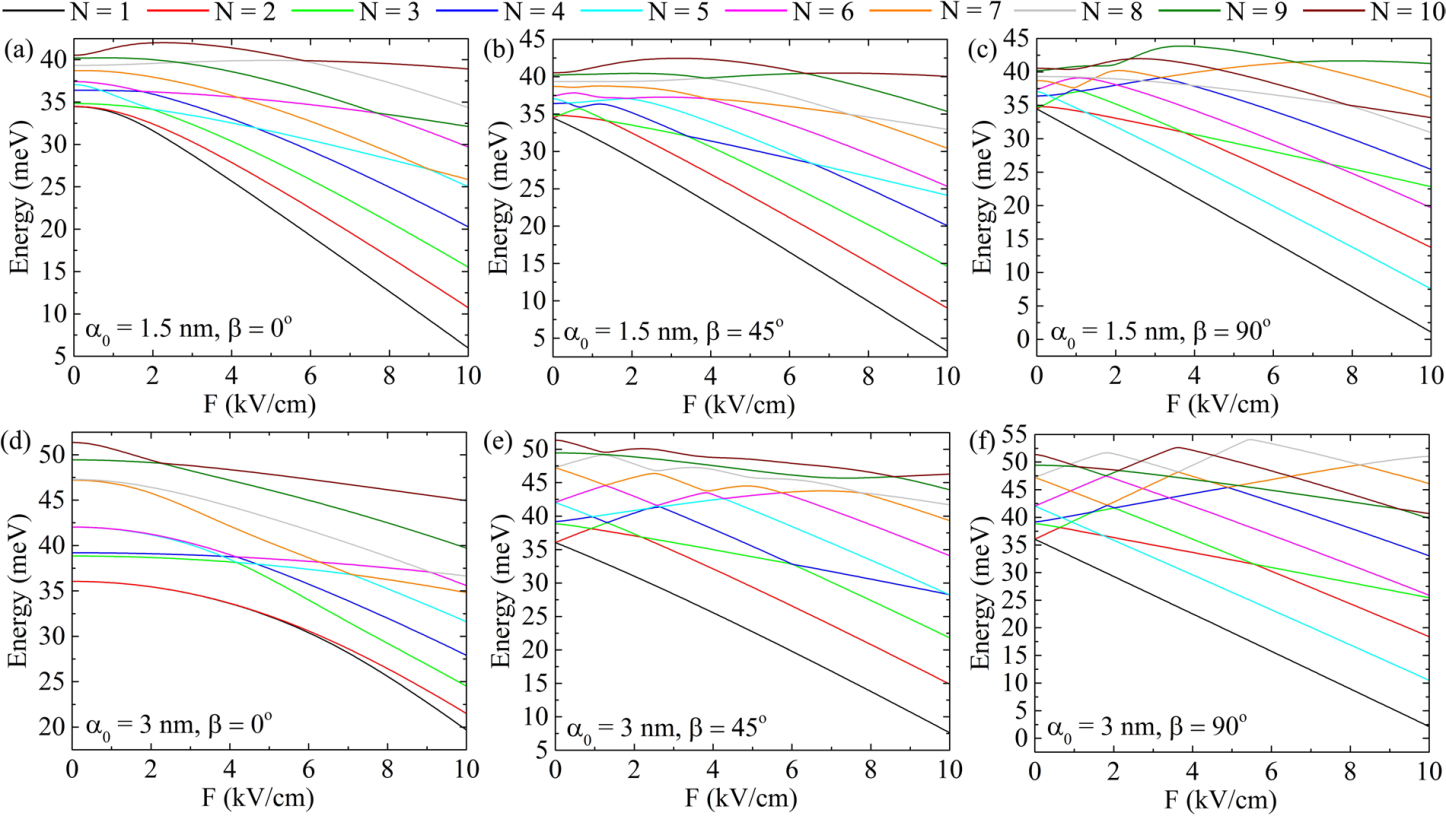
Stark effect on laser-dressed energy levels. The following pairs of *α*_0_ and *β* parameters are considered: (**a**) *α*_0_ = 1.5 nm, *β* = 0°; (**b**) *α*_0_ = 1.5 nm, *β* = 45°; (**c**) *α*_0_ = 1.5 nm, *β* = 90°; (**d**) *α*_0_ = 3 nm, *β* = 0°; (**e**) *α*_0_ = 3 nm, *β* = 45° and (**f**) *α*_0_ = 3 nm, *β* = 90°.

In this section, we consider static electric field effect on already laser-dressed CDQRs. Figure 6 explores the influence on the energy levels of the electric field applied in different directions. The direction is defined by $\beta =\angle (\hat{{\bf{u}}},{\hat{{\bf{e}}}}_{x})$ angle, where $\hat{{\bf{u}}}$ and ${\hat{{\bf{e}}}}_{x}$ are unit vectors of electric field and laser field polarization, respectively. In case of an electric field directed along the *x*-axis (*β* = 0°) quadratic Stark effect^49^ is observed for both values of *α*_0_ = 1.5 nm; 3 nm, and all the energy levels decrease as a reason of effective confining potential tilting demonstrated in Fig. 1(b). In addition, since $\hat{{\bf{u}}}$ vector direction is also symmetrical one for the only laser field affected potential in Fig. 1(a), related wave functions have symmetry or antisymmetry with respect to the *x*-axis, and energy spectrum can reveal both crossing and anti-crossing points. On the other hand, for *β* = 45° case the direction of $\hat{{\bf{u}}}$ does not follow the symmetry of *V*_d_(**r**_⊥_) potential energy. This implies that the related wave functions do not have any distinct symmetry or antisymmetry. Thus, the energy levels cannot express any crossing behavior. If the direction of electric field is perpendicular to ${\hat{{\bf{e}}}}_{x}$ (*β* = 90°) energy levels start to become linear functions of *F*, that are more clearly observed for *α*_0_ = 3 nm, or in other words, when the anisotropy of the confining potential is greater. There are experimental studies that pointed out the importance of anti-crossing features that can be used to measure the degeneracy in coupled quantum systems. For instance, studies in refs^50,51^ demonstrated the possibility to measure the tunnel splitting in a double QD charged with a single electron. Also, by magnetophotoluminescence spectroscopy the existence of a hole-spin-mixing term directly related to the anti-crossings in the excitonic spectrum was obtained in electric field influenced InAs QD molecules (see ref.^52^). In the context of the mentioned works, we show that one can effectively control the anti-crossings in the energy spectrum of CDQRs with electric field once the anisotropy is achieved.

**Figure 7:**
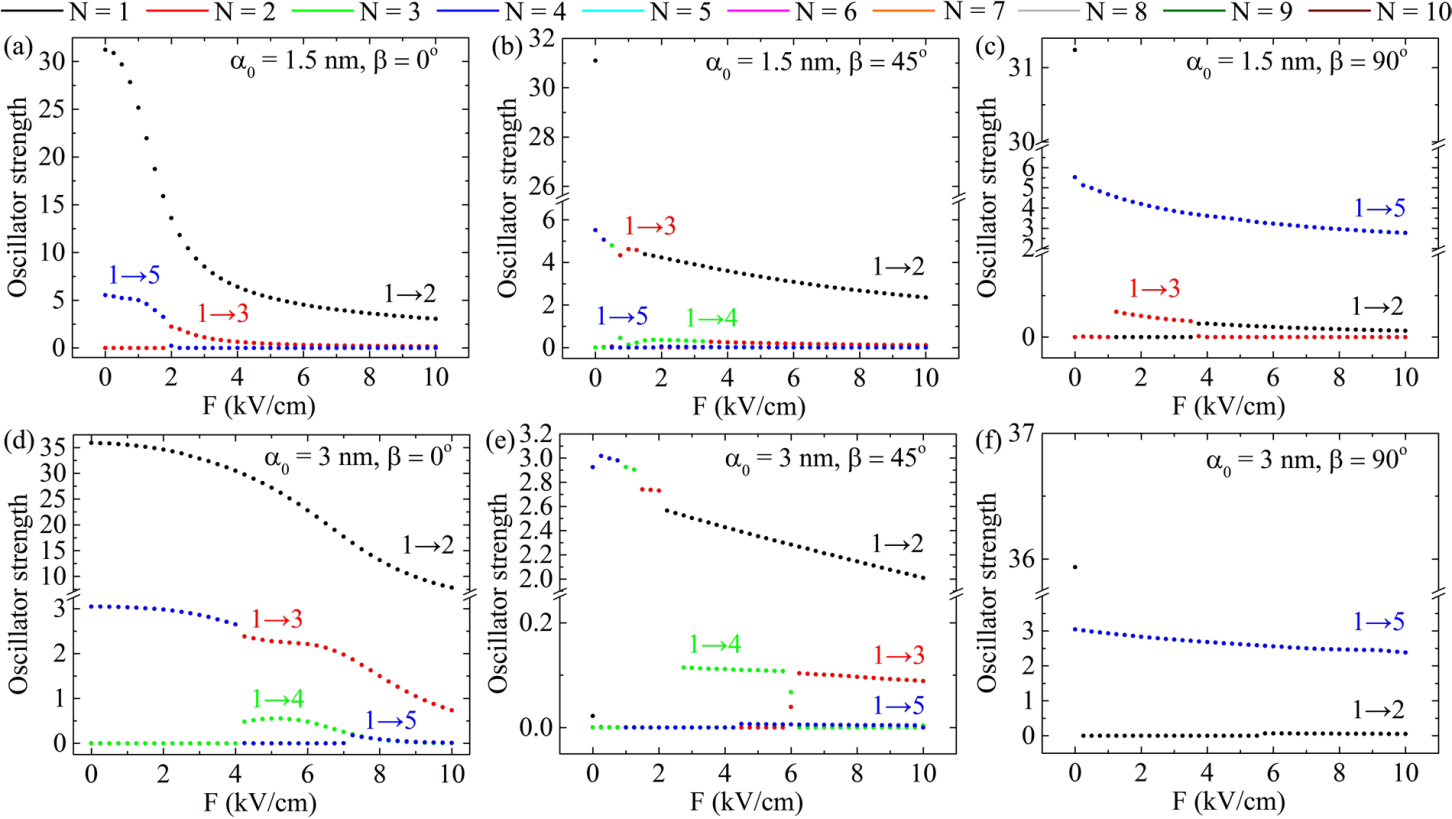
The oscillator strengths of the most intensive intraband transitions. The following pairs of *α*_0_ and *β* parameters are considered: (**a**) *α*_0_ = 1.5 nm, *β* = 0°; (**b**) *α*_0_ = 1.5 nm, *β* = 45°; (**c**) *α*_0_ = 1.5 nm, *β* = 90°; (**d**) *α*_0_ = 3 nm, *β* = 0°; (**e**) *α*_0_ = 3 nm, *β* = 45° and (**f**) *α*_0_ = 3 nm, *β* = 90°.

Besides that, the electric field can serve as a potential tool to manipulate the optical response of the CDQR system. In the presence of electric field all the transitions are allowed, since *M*_if_ matrix element never becomes zero. The oscillator strengths^27,53^

8 $${O}_{{\rm{if}}}=\frac{2m}{\hslash }{{\rm{\Delta }}}_{{\rm{if}}}{|{M}_{{\rm{if}}}|}^{2}$$

of the most intensive transitions are demonstrated in Fig. 7. The results are respectively related to the energy spectra in Fig. 6. While 1 → 2 is observed as the most probable transition one for all the values of *F* in *β* = 0° case, the appearance of a non-zero angle changes the scenario. For *α*_0_ = 1.5 nm and *β* = 45° values, although the highest value is obtained for *O*_12_ at *F* = 0 (Fig. 7(b)), in [0.25 kV/cm, 1.25 kV/cm] range probabilities of other transitions are prevailing. Further increase of *F* makes *O*_12_ the biggest in Fig. 7(b). In the situation with the same *β* = 45° but greater *α*_0_ = 3 nm given in Fig. 7(e) *O*_13_, *O*_14_ and *O*_15_ depict the most probable transitions in [0, 2 kV/cm]. Finally, cases of electric field perpendicularly (*β* = 90°) to the direction of laser field polarization vector is explored in Fig. 7(c) and (f). Now 1 → 5 is the most probable one for all the considered values of *F*. Only in the absence of electric field 1 → 2 turns out to be the most intensive one.

**Figure 8:**
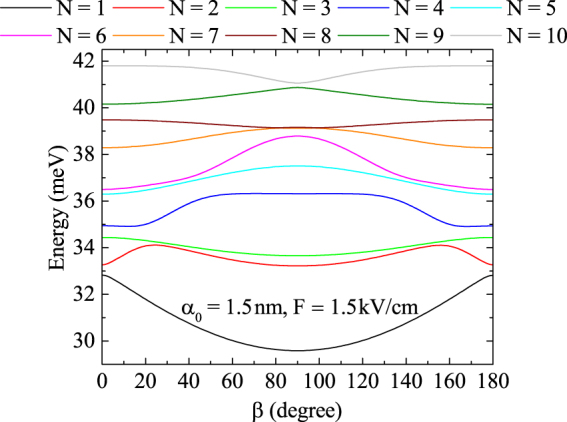
Energy spectrum dependence on *F* = 1.5 kV/cm electric field direction for *α*_0_ = 1.5 nm.

Figure 8 the combined influence of laser and electric fields by changing the direction of electric field of *F* = 1.5 kV/cm strength and keeping the polarization vector ${\hat{{\bf{e}}}}_{x}$ of laser field fixed is considered. As Fig. 8 shows, the energy levels mostly have extrema for *β* = 90°, with the exception of the second excited energy level that has maxima at *β* = 24° and *β* = 156° and minimum at *β* = 90°, and the fourth one that together with the minima at *β* = 13° and *β* = 167° are almost constant in [72°, 108°] interval. The appearance of the extrema and the region of invariance can be attributed to the complex distribution of electron cloud throughout the variation of *β*. In addition, the observed symmetry with respect to the *β* = 90° point, is caused by the mirror symmetry of the dressed potential in Fig. 1(a) with respect to the *x*− and *y*− axis, which means that at angles *β* and 180° − *β* electric field affects identically.

**Figure 9:**
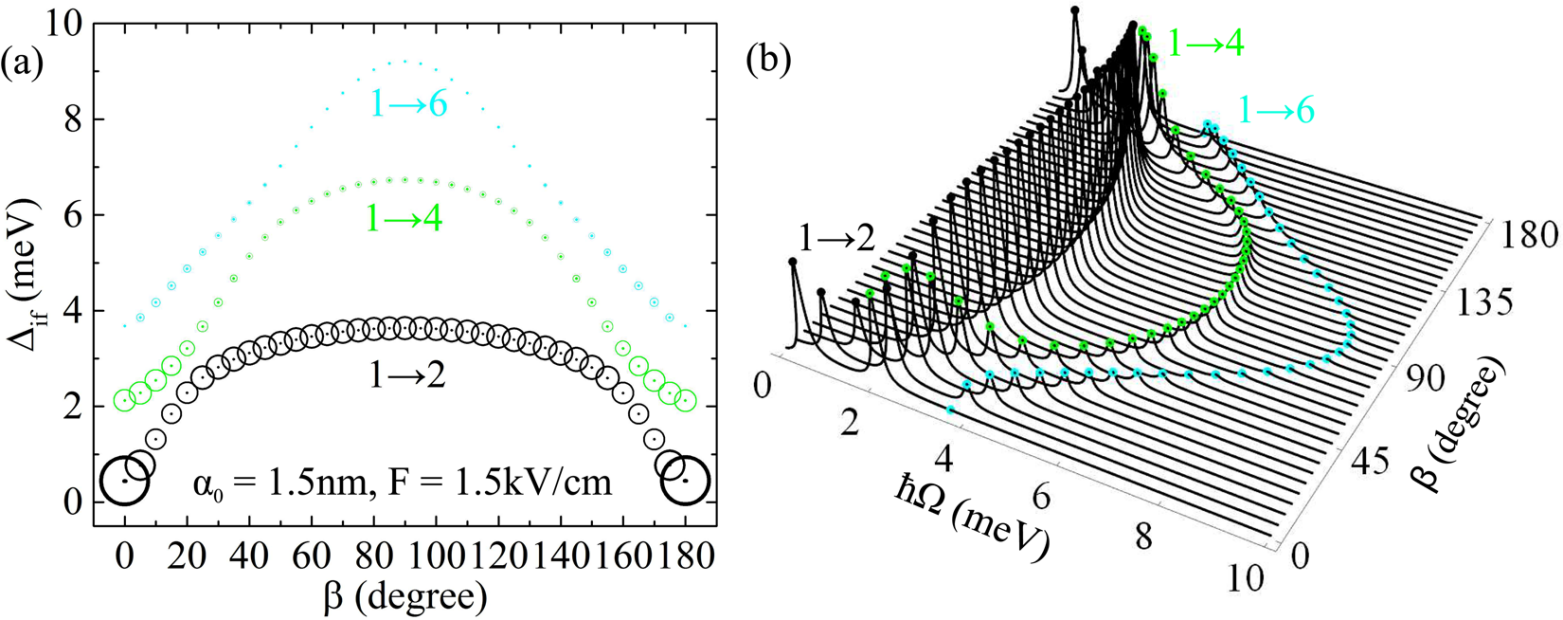
Δ_if_ energy dependence on *β* (**a**) and corresponding absorption coefficient (in arbitrary units) dependence on incident photon energy $\hslash {\rm{\Omega }}$ for different directions (defined by *β*) of *F* = 1.5 kV/cm electric field (**b**). Laser field parameter is fixed to *α*_0_ = 1.5 nm and the area of the circles in Fig. 9(a) is proportional to the respective dipole matrix element |*M*_if_|^2^.

And finally, Fig. 9(a) shows all the most intensive transitions to undergo blueshift of the absorption spectrum in the [0°, 90°] interval and redshift in [90°, 180°]. The related absorption coefficient is demonstrated in Fig. 9(b) considering different values of *β*. In this case, at the beginning of *β* variation and close to *β* = 180° only 1 → 4 transition has absorption coefficients of values comparable with 1 → 2 one, but for the other *β*, the latter transition has the biggest absorption coefficient.

## Final Remarks

We have demonstrated that the electronic and optical properties of CDQR system can be readily controlled with THz laser and static electric fields. Particularly, it is calculated that the intense THz laser field permits to study double QRs of different geometries in a single sample of CDQRs, that is an important finding for modeling of experimental studies. In addition, by changing the characteristic parameter *α*_0_ of the laser field, one can come to a new set of degenerated levels in laser-dressed CDQR and manage the distribution of electron cloud between the rings. The impact of inter-ring barrier width *L*_B_ variation on electron PD in the rings shows that delocalization of PD from the inner to the outer ring does not depend on the fixed value of *L*_B_/*α*_0_ ratio.

The selection rule that defines the intraband transitions of the circularly polarized light in only laser field influenced CDQR system are shown to allow only the transitions from the ground state to the excited states that do not have antisymmetry or symmetry with respect to both coordinate axes. Also, with the augmentation of *α*_0_ both the blue- and redshifts of the absorption spectrum are observed.

The addition of a static electric field on the energy spectrum results in linear and quadratic Stark effects, caused by the anisotropic modification of confining potential by the laser field. Linear Stark effect is observed for the electric field perpendicular to laser field polarization and is more pronounced for the larger anisotropy of the confining potential. For the quadratic Stark effect, the direction parallel to laser field is favorable. Also, the electric field direction drastically changes the crossing and anti-crossing behaviors of the energy levels. Moreover, electric field removes the laser field-induced selection rule and allows all the intraband transitions.

Besides that, it is shown that the electric field influence on the laser-dressed CDQRs allows to readily control the anti-crossings in the energy spectrum. In addition, if the electric field is parallel to laser polarization the biggest oscillator strength (absorption intensity) has 1 → 2 transition. In case of a perpendicular direction of the electric field, 1 → 5 transitions have the biggest intensity. Electric field direction changing also affects the absorption spectrum, making mainly blueshifts in [0°, 90°] and redshifts in [90°, 180°] orientations. It is worth to note that the THz laser field can in principle control any anisotropic (induced by the geometry, effective mass, defects, etc.) properties of the CDQR nanostructure. We believe that the results are useful and will open up new possibilities to the improved design and characterization of new devices based on CDQR, such as THz detectors, efficient solar cells, photon emitters, to cite a few.
